# Psychiatric advance directives facilitated by peer workers among people with mental illness: economic evaluation of a randomized controlled trial (DAiP study)

**DOI:** 10.1017/S2045796023000197

**Published:** 2023-04-25

**Authors:** S. Loubière, A. Loundou, P. Auquier, A. Tinland

**Affiliations:** 1 Department of Clinical Research and Innovation, Support Unit for Clinical Research and Economic Evaluation, Assistance Publique – Hôpitaux de Marseille, Marseille, France; 2 Health Service Research and Quality of Life Center (UR 3279), Aix-Marseille University, School of Medicine, Marseille, France; 3 Department of Psychiatry, Assistance Publique – Hôpitaux de Marseille, Marseille, France

**Keywords:** health economics, mental health, other psychosocial techniques/treatments, psychiatric services, randomized controlled trials

## Abstract

**Aims:**

We aimed to assess the cost-effectiveness of psychiatric advance directives (PAD) facilitated by peer workers (PW-PAD) in the management of patients with mental disorders in France.

**Methods:**

In a prospective multicentre randomized controlled trial, we randomly assigned adults with a Diagnostic and Statistical Manual of Mental Disorders, fifth edition diagnosis of schizophrenia, bipolar I disorder or schizoaffective disorders, who were compulsorily hospitalized in the past 12 months, to either fill out a PAD form and meet a peer worker for facilitation or receive usual care. We assessed differences in societal costs in euros (€) and quality-adjusted life-years (QALYs) over a year-long follow-up to estimate the incremental cost-effectiveness ratio of the PW-PAD strategy. We conducted multiple sensitivity analyses to assess the robustness of our results.

**Results:**

Among the 394 randomized participants, 196 were assigned to the PW-PAD group and 198 to the control group. Psychiatric inpatient costs were lower in the PW-PAD group than the control group (relative risk, −0.22; 95% confidence interval, [−0.33 to −0.11]; *P* < 0.001), and 1-year cumulative savings were obtained for the PW-PAD group (mean difference, −€4,286 [−4,711 to −4,020]). Twelve months after PW-PAD implementation, we observed improved health utilities (difference, 0.040 [0.003–0.077]; *P* = 0.032). Three deaths occurred. QALYs were higher in the PW-PAD group (difference, 0.045 [0.040–0.046]). In all sensitivity analyses, taking into account sampling uncertainty and unit variable variation, PW-PAD was likely to remain a cost-effective use of resources.

**Conclusion:**

PW-PAD was strictly dominant, that is, less expensive and more effective compared with usual care for people living with mental illness.

## Introduction

Involuntary treatment and care is common and increasing in high-income countries, with variation by country (Wasserman *et al*., 2020). Compulsory hospital admissions, whether or not associated with other coercive measures, are important causes of trauma and negative treatment outcomes among people with mental disorders. Several studies show that these episodes of deprivation of liberty constitute a very negative experience (Nyttingnes *et al*., 2016; Sibitz *et al*., 2011), affecting quality of life (Swanson *et al*., 2003), with little evidence of effectiveness in terms of health status, social functioning and use of services (Hofmann *et al*., 2022). The overall cost of compulsory hospitalizations has scarcely been studied (Venturini *et al*., 2017).

Different models of interventions to reduce compulsory admissions have been developed. Of these, psychiatric advance directives (PADs) or joint crisis plans (JCPs) are written documents that allow adults with mental illness and with temporary decision-making incapacity to state their will and preferences in advance, to be applied if further mental health crises impair their decision-making capacity (Henderson *et al*., 2008).

Behind this common objective, PADs and JCPs differ in several ways, including their legal force or with whom they are fulfilled (Atkinson *et al*., 2003). On the one hand, the specificity of PADs is that they emphasize that they are legal documents, on the other hand, JCPs rather emphasize that they are on an agreement signed by the person, the healthcare professionals and possibly the relatives. Authors have highlighted profound differences in the way people’s autonomy is represented in JCPs and PADs: more absolute for PADs advocates and more assisted for JCPs advocates (Ambrosini and Crocker, 2010). Despite these differences, PADs and JCPs have so much in common that they are routinely categorized as a similar intervention and analysed together in systematic reviews, which all rank them among the most effective interventions for reducing compulsory admission (Barbui *et al*., 2021; Bone *et al*., 2019; DeJong *et al*., 2016). A meta-analysis evaluated that PADs and JCPs reduced compulsory admission by 25% (Molyneaux *et al*., 2019).

Inherently, PADs promote user’s involvement and dialogue (Murray and Wortzel, 2019). The benefits in terms of autonomy of PADs were at first only theoretical but have gradually gained ground as studies have shown that PADs improve user involvement, empowerment and recovery; the therapeutic alliance and integration of care, but these positive results are still low in evidence (Nicaise *et al*., 2013). Recently, a randomized controlled trial that we conducted showed that PADs facilitated by peer workers were associated with fewer symptoms (effect size [95% confidence interval, CI]: −0.20 [−0.40 to 0.00]), higher empowerment (0.30 [0.10 to 0.50]) and higher recovery (0.44 [0.24 to 0.65]) compared to the control group (Tinland *et al*., 2022).

The model of PADs that we have experimented in this trial had the originality of being facilitated by peer workers, that is, people with personal experiences of mental distress who are trained to assist users in filling their PAD statement and sharing it with relatives and psychiatrists. We observed that this model of PADs was associated with a significant reduction of over 32% in the proportion of compulsory hospitalized people (main criterion of our trial) (Tinland *et al*., 2022). As in the Molyneaux’ meta-analysis, we found a less clear effect on voluntary hospitalizations and on the total number of hospital admissions. The latter result raises the question of the cost-effectiveness of PADs facilitated by peer workers.

To our knowledge, and despite its societal importance, PAD interventions have received little attention in terms of costs, and only two of the randomized trials on the topic have explored this parameter, exclusively in the UK (Barrett *et al*., 2013; Flood *et al*., 2006), and the difference in costs was not significant. Despite counting its effectiveness among people with mental illness, it is unclear whether reducing compulsory admissions results in cost savings or a shift in care to non-compulsory admissions amounting to the same length of stay. The main objective of this study was to conduct an economic evaluation of psychiatric advance directives facilitated by peer workers (PW-PAD) as part of a longitudinal randomized clinical trial for people with severe mental illness.

## Methods

### Trial design

The ‘Peer-Worker-Facilitated Psychiatric Advance Directive’ study (DAiP) was a multicentre randomized controlled trial conducted in seven mental health facilities of three cities in France (Lyon, Paris and Marseille) between January 2019 and June 2021. Forty treating psychiatrists checked the eligibility criteria and referred eligible participants to research assistants, who validated inclusion criteria, obtained written consent and conducted interviews at inclusion, 6 and 12 months. According to the principle of ‘sector’, which has organized most public psychiatric care in France since the 1960s, the participating psychiatrists were both inpatient and outpatient (the same team ensures continuity). Only six of them were strictly outpatient. All psychiatrists at each participating mental health facility were fully informed about the study at the time of its implementation. No special training or incentives were given to participating psychiatrists. The follow-up period was 12 months after an 18-month recruitment.

The study was registered on Clinicaltrials (NCT03630822). The study was conducted in compliance with the Declaration of Helsinki, sixth revision; Good Clinical Practice guidelines and local regulatory requirements. The trial was approved by the local ethics committee (2018-A00146-49).

### Population

The inclusion criteria were being over 18 years of age; with experience of involuntary admission to hospital within the past 12 months; with a diagnosis of schizophrenia, bipolar I disorder or schizoaffective disorders according to Diagnostic and Statistical Manual of Mental Disorders, fifth edition criteria; with decision-making capacity assessed by a psychiatrist according to the MacArthur Competence Assessment Tool for Clinical Research (Appelbaum and Grisso, 1995) and with French government health insurance. The exclusion criteria included being considered unable to provide informed consent and being under the highest level of guardianship. At the time of inclusion, most participants were discharged from the hospital; nevertheless, a few participants were included as inpatients.

### Randomization

Participants were randomly assigned using a web-based system at a 1:1 ratio. Treating clinicians, participants and research assistants were aware of the assigned randomization group.

### Intervention group: PW-PAD

All PW-PAD participants received the PW-PAD document from research assistants, consisting of future treatment and support preference options, description of early signs of relapse and coping strategies (English translation in the Supplementary material). Depending on their wishes, the PW-PAD participants could (i) meet a peer worker in a place of their choice; (ii) be supported by this peer worker in drafting the PAD document with as many meetings as necessary and (iii) be supported by the peer worker during the sharing of PAD with the healthcare agent and the psychiatrist. Peer support workers were recruited specifically for this study and trained at a Recovery college (Centre de Formation au Rétablissement – CoFoR). Regular exchanges were organized between them (intervisions) and with the whole research team, both remote and face-to-face.

Hard PADs were stored by the health worker or psychiatrist depending on the participant’s choice and in electronic format if available and requested. In case of crisis, reporting of the existence of PADs was done by patients, their entourage or informed caregivers.

### Control group

People assigned to the control group were treated as usual. Depending on the person’s needs, the usual treatment includes psychological, pharmaceutical and social support. In France, especially at the time of this study, JCPs were only used locally by a few pioneering teams, and there was very little chance that people in the control group would access to crisis plans. Nonetheless, they received comprehensive information about the PAD concept during the inclusion step and were free to fill out a PAD, but with no connection to the study’s peer worker. Figure S1 (see Supplementary material) reports the number of PAD (PW-PAD or other) completion and PAD use in each group.

### Cost-effectiveness analysis

We performed a cost-utility analysis based on the societal perspective, including hospital, outpatient and community care, and productivity losses due to illness. Incremental cost-effectiveness ratios (ICERs) were expressed in terms of costs per quality-adjusted life-year (QALY) gained, in accordance with Consolidated Health Economic Evaluation Reporting Standards (CHEERS) and the French National Authority for Health (HAS) guidelines for economic evaluation (HAS, 2020; Husereau *et al*., 2022).

### Effectiveness measure

Utilities for health states were assessed using the EuroQol-5 Dimensions, three-level version (EQ-5D-3L) (Brooks, 1996; Chevalier and de Pouvourville, 2013). This questionnaire is a validated questionnaire that assesses a participant’s health status through five dimensions: mobility, personal care, routine occupations, pain and discomfort and anxiety and depression. Each dimension has three levels: no problems, some problems and severe problems. The index score ranges from 0 (worst utility) to 1 (best utility). Quality-of-life measures at baseline, 6 and 12 months were summed as QALYs using an area under the curve approach (Hunter *et al*., 2015; Husereau *et al*., 2022) and compared between the PW-PAD and control groups.

### Costs measure

We considered all direct and indirect healthcare costs in relation with care management during the follow-up. Resource use data were retrieved from two sources: hospital-based administrative databases and patients’ self-reported measures. Resource use for each collaborative hospital was retrieved for all randomized patients. To measure ambulatory and community care, and at the margin to supplement hospital registry data, interview grids were built to assist participants in reporting individual and prospective resource use, based on previous studies of service use among people with mental illness (Latimer *et al*., 2017; Loubière *et al*., 2020). Resource utilization included those relating to the intervention, including training of peer workers and time spent for PAD support, visits to the emergency department (ED), psychiatric hospital admissions and total number of days at hospital, as well as outpatient care. These latter costs were assessed through consultations with general practitioners, referring psychiatrists and other specialists. Indirect costs were investigated based on the number of days of work absenteeism and compared between groups where relevant.

Unit costs for hospital resources were estimated using data from the French National Hospital Database (https://www.atih.sante.fr/). The training of peer workers consisted in two half-day sessions per week for 6 weeks, with an estimated cost of €1,215. We observed the real cost of recruiting a peer worker at each site, either a full-time or part-time contract over the duration of the study. The average salary scales at the participating facilities were used to estimate the monthly salary of the peer workers. No overhead costs were charged to the intervention: the office room and equipment (i.e., computer and telephone) were already present and shared with the care/administrative team. In the city of Paris (i.e., for two health facilities), transportation costs were taken into account (intra-city transportation card). For outpatient and community care costs, national tariffs were used (Source: National databases for medical and paramedical acts). All resources were valued in 2019 euros (see Supplementary Table S1) and discounting was not applied.

### Statistical analyses

An intention-to-treat analysis was conducted in the present study and included all participants randomized in each group whether or not they received the intervention or were lost to follow-up. To detect a reduction of 30% in the rate of compulsory admissions to psychiatric hospitals during the 12-month follow-up, the planned sample was 200 per group, i.e., 400 in total. This number of subjects allowed for a minimum cost difference of €320 with a standard deviation of 1,000, at a statistical power of 90%.

Missing data were addressed using multiple imputations (van Buuren, 2007), under the assumption of missing at random (Ware *et al*., 2012). Markov chain Monte Carlo multiple imputation was used, which creates multiple ‘complete’ datasets by predictions for each missing value. Fifty imputed datasets were implemented using Multivariate Imputation by Chained Equations and mitools R packages. We ran a sensitivity analysis for only the observed data (excluding missing data).

A mixed model for longitudinal utility values was used to control for potential bias due to intra-patient correlated data and the existence of co-variates influencing quality of life. Between-group differences in service use and total costs were estimated using generalized estimating equations (GENLIN function) applying a Poisson distribution with a link log due to skewness. Mean differences and beta coefficients with 95% CI were provided.

We used non-parametric bootstrapping (with 5,000 replications) to resample observations. The bootstrap results were combined to calculate the mean values for costs and utilities, and the SEs for the imputed values were used to calculate 95% CIs. The incremental cost per QALY gained was then calculated as mean incremental costs divided by mean incremental QALYs and reported where relevant. Uncertainty in the cost-effectiveness results was analysed using both univariate deterministic and probabilistic sensitivity analyses.

Statistical analyses were performed using R Studio (R version 4.0.2, RStudio, Inc., Massachusetts, USA) and TreeAgePro 2019.

## Results

A total of 394 participants were included in the analysis: 196 were assigned to the intervention group and 198 to the control group. Interviews at the 12-month follow-up were completed for 127 (65%) in the PW-PAD group (four participants withdrew) and 139 (70%) in the control group (including five withdrawals) (see details in Supplementary Figure S2). Baseline characteristics and primary clinical outcomes are reported in Table 1. Gender, age, comorbidities and experience of previous hospital admissions were well balanced between the two groups. PW-PAD participants showed significantly improved self-reported mental symptoms, recovery and empowerment scores over the 12-month follow-up compared with the control group.

**Table 1.** Socio-demographic and clinical characteristics of participants (*N* = 394)

Characteristics	PW-PAD goup (*n* = 196)	Control group (*n* = 198)
Gender, *N* (%)		
Men	127 (64.8)	112 (56.6)
Age mean (SD), y	37.4 (11.7)	41.0 (12.7)
Median (IQR)	36 (28–44)	40 (31–49)
Nationality, *N* (%)		
French	184 (93.9)	180 (91.8)
Education, *N* (%)		
Less than high school (<bac)	57 (29.2)	75 (37.9)
Completed or postsecondary school	138 (70.8)	123 (62.1)
Marital status, *N* (%)		
Single	132 (67.3)	128 (64.6)
Married/partnered	38 (19.4)	35 (17.7)
Divorced/separated/widow	26 (13.3)	35 (17.7)
Work activity, *N* (%)		
Yes	33 (18.8)	37 (19.9)
EPICES score mean (SD)	40.6 (19.9)	42.8(20.9)
Median (IQR)	40.8 (24–57)	44.6 (26–59)
DSM-5 diagnosis, *N* (%)		
Bipolar I disorder	66 (33.8)	73 (36.9)
Schizophrenia	86 (44.1)	92 (46.5)
Schizoaffective disorders	43 (22.1)	33 (16.7)
Alcohol dependence, *N* (%)		
Yes	6 (3.4)	6 (3.5)
Substance dependence, *N* (%)		
Yes	22 (12.6)	24 (13.6)
With at least one somatic comorbidity, *N* (%)		
Yes	120 (61.2)	137 (69.2)
CGI score mean (SD)	4.1 (1.2)	4.3 (1.1)
Median (IQR)	4.0 (3–5)	4.0 (4–5)
Number of admissions in the previous year, mean (SD)	1.5 (0.9)	1.4 (0.8)
1	132 (67.3)	148 (75.5)
2	45 (23.0)	37 (18.9)
≥3	17 (8.7)	11 (5.6)
Clinical outcomes		
MCSI score, mean (SD)	11.49 (11.91)	13.87 (10.99)
ES score, mean (SD)	16.80 (26.32)	10.20 (16.04)
RAS score, mean (SD)	72.60 (14.13)	65.55 (13.92)

PW-PAD: peer worker–facilitated psychiatric advance directive; SD: standard deviation; IQR: interquartile range; DSM-5: Diagnostic and Statistical Manual of Mental Disorders, fifth edition; CGI: Clinical Global Impression scale.

Recovery was assessed using the Recovery Assessment Scale (RAS) (Corrigan *et al*., 2004), which measures various aspects of recovery from the perspective of the consumer, with a particular emphasis on hope and self-determination. This self-administered instrument comprises 24 items, exploring five domains: personal confidence and hope, willingness to ask for help, goal and success orientation, reliance on others and no domination by symptoms. A higher score indicates better recovery.

Mental health symptomatology was assessed using the self-report modified Colorado Symptom Index (MCSI; Conrad *et al*., 2001). The MCSI contains 14 items, which evaluate how often in the past month an individual has experienced a variety of mental health symptoms, including loneliness, depression, anxiety and paranoia. Higher scores indicate a greater likelihood of mental health problems.

Empowerment was assessed using the Empowerment Scale (ES) (Rogers *et al*., 1997). The ES comprises 28 items, split into five dimensions: community activism and autonomy, self-esteem and efficacy, optimism and control over the future, righteous anger and power and powerlessness. The index score is 0 to 100, where higher scores correspond to higher empowerment.

### Effectiveness

The mean utility at baseline (standard error [SE]) for the PW-PAD and control groups were, respectively, 0.798 (0.015) and 0.757 (0.020) (*P* = 0.101) (Table 2). Participants in the PW-PAD group reported higher health utilities at month 12 (0.814 vs. 0.755; *P* = 0.017). From baseline to 12 months of follow-up, health utilities improved more in the PW-PAD group than in the control group (mean difference, 0.040; 95% CI, 0.003–0.077; *P* = 0.032). Based on complete data, the mean difference in health utilities was 0.045 (95% CI, 0.002–0.088; *P* = 0.039) (see Supplementary Table S2).

**Table 2.** Change in health utilities score (EQ5D-3L) during follow-up in the DAiP trial

	Baseline		Month 6		Month 12		
	PW-PAD group	Control group	PW-PAD group	Control group	PW-PAD group	Control group	Mean difference^b^ (95% CI)
Mean utility	0.798	0.757	0.774	0.753	0.814	0.755	0.040 (0.003–0.077)
Standard error	0.015	0.020	0.019	0.021	0.017	0.018	
*P* value^a^	0.101		0.459		*0.017*		*0.032*^b^

PW-PAD: peer worker–facilitated psychiatric advance directive; SE: standard error; 95% CI: 95% confidence interval.

a *P*-values at each time were provided by *t*-test analysis (independent samples test) based on imputed data.

b Mixed linear models (MIXED) for repeated-measure analyses were applied, using a restricted maximum likelihood approach for variance estimation, with a repeated variable ln(*t* + 1), where *t* is the time from baseline. An unstructured covariance matrix for repeated measures was used. The interaction between group and time was tested and was not kept because none achieved statistical significance.

Values in italic indicate a statistically significant difference from the group variable (PW-PAD vs. control groups)

Three (0.76%) patients died during the 12-month follow-up: two (1.01%) patients in the TAU group and one (0.51%) patient in the PW-PAD group.

### Service use and costs

Details of service resource use and mean costs are provided in Table 3. Table S2 (see Supplementary material) reports total psychiatric hospital admissions and length of stay in both compulsory and voluntary settings. The PW-PAD group experienced less psychiatric hospital days over 1 year compared to the control group (45.4 vs. 57.1; *P* = 0.026). No significant differences were found in the mean number of ED visits or consultations (*P* = 0.056 and *P* = 0.309; respectively). Similarly, the rates of patients having working activity at the end of follow-up did not show significant difference between the PW-PAD and control groups (36.6% vs. 28.2%; *P* = 0.184). No significant differences were found in the mean number of days off work between the PW-PAD and control groups (3.75 vs. 2.54 days; *P* = 0.383).

**Table 3.** Mean healthcare utilization and costs at 12 months of follow-up

	Utilization				Costs		
	PW-PAD group (*n* = 196)	Control group (*n* = 198)			PW-PAD group (*n* = 196)	Control group (*n* = 198)	
Outcomes	Mean (SE)	Mean (SE)	Mean difference (95% CI)		Mean (SE)	Mean (SE)	Relative risk (95% CI)^a^
No. of days in psychiatric hospitalization	45.41 (3.28)	57.08 (4.09)	*−11.67 (−21.95 to –1.39)*	Total inpatient costs	20,127.25 (867.27)	25,062.54 (800.41)	*−0.22 (−0.33 to –0.11)*
No. of emergency department visits	1.61 (0.36)	0.90 (0.23)	0.71 (−0.02 to 1.44)	Emergency department costs	278.75 (58.67)	158.21 (36.32)	*0.59 (0.11 to 1.08)*
No. of ambulatory visits	13.79 (1.05)	12.35 (0.93)	1.44 (−1.33 to 4.20)	Ambulatory costs	1,290.95 (28.48)	1,168.66 (19.29)	*0.10 (0.05 to 0.16)*
				Intervention costs	399.66 (3.63)	–	–
				Total costs	22,094.27 (873.36)	26,382.39 (836.15)	*−0.18 (−0.28 to –0.08)*

PW-PAD: peer worker–facilitated psychiatric advance directive; SE: standard error; 95% CI: 95% confidence interval.

a A Poisson distribution with a link log was used.

Generalized linear models were used to address mean difference and 95% CI for the groups.

Values in italic indicate a statistically significant difference in pooled imputation dataset from the group variable (PW-PAD vs. control groups), *P* < 0.05.

Compared with the control group, PW-PAD exhibited a statistically significant cost difference in total costs (€22,094.27 vs. € 26,382.39; *P* = 0.001) (Table 4).

**Table 4.** Mean and incremental costs and QALYs for patients receiving PW-PAD versus usual care

	PW-PAD group, mean (95% CI)	Control group, mean (95% CI)	Incremental, mean (95% CI)
QALYs	0.789 (0.783–0.794)	0.746 (0.740–0.752)	0.045 (0.040–0.046)
Costs	22,095 (21,498–24,436)	26,382 (25,695–27,981)	−4,286 (−4,711 to –4,020)
ICER			−99,977 (−113,378 to −91,035)

PW-PAD: peer worker–facilitated psychiatric advance directive; QALY: quality-adjusted life-years; CI: confidence interval.

### Cost-effectiveness

The incremental benefit of PW-PAD versus control group was 0.045 QALY (95% CI, 0.040 to 0.046); the incremental cost was −€4,286 (95% CI, −4,711 to −4,020) (Table 4). The cost-effectiveness analysis showed that the PW-PAD intervention was strictly dominant, that is, less expensive and more effective compared to the usual care.

**Fig. 1.**
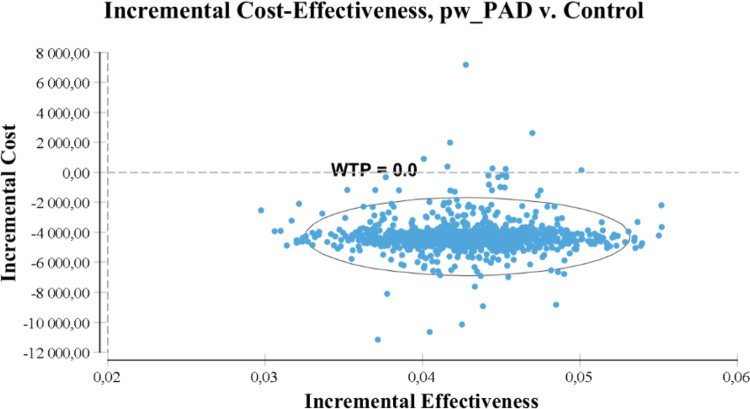
Cost-effectiveness plane associated with the 12-month cost per QALY analysis.

PW-PAD: peer worker–facilitated psychiatric advance directive; QALY: quality-adjusted life year; WTP: willingness to pay.

The bootstrap distribution of the ICER showed that 100% of the 5,000 replicates of ICER were located in the lower-right quadrant of the scatterplot plan (Fig. 1).

The graph contains axes that represent the incremental cost (*y*-axis) and incremental effectiveness (*x*-axis). Each point in the graph represents the incremental cost and incremental effectiveness values (PW-PAD vs. control) from a single recalculation from the database.

The willingness to pay (WTP), or ICER threshold, is used as the slope of a line intersecting the origin of the plot. The WTP line in the graph intersects points having the specified ICER value, and the region below and to the right of the line includes points where the intervention/PW-PAD is more cost-effective than the usual care/control. The ellipsis shows the 95% CI.

### Sensitivity analyses

**Fig. 2.**
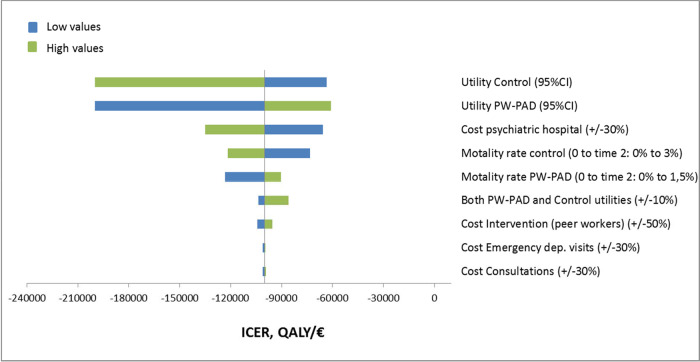
One-way sensitivity analysis of cost-effectiveness associated with PW-PAD intervention.

PW-PAD: peer worker–facilitated psychiatric advance directive; QALY: quality-adjusted life year; CI: confidence interval; ICER: incremental cost-effectiveness ratio.

Sensitivity analyses with complete cases yielded similar results to imputed datasets (Table S3). The tornado diagram (Fig. 2) indicated that cost-effectiveness was most strongly affected by utility values altered over their 95% CI and inpatient costs varying from ±30%. This was followed by the mortality rates in the PW-PAD and control groups. Regardless of the change in these parameters, the higher effectiveness and cost savings were maintained for PW-PAD. In addition, increasing or decreasing the costs of the intervention by 50% had less effects on the ICER.

Taking sampling uncertainty into consideration, the cost-effectiveness acceptability curve for the base-case analysis (Figure S3) shows a 100% probability that PW-PAD was cost-effective by comparison with usual care at the threshold of €1,000 WTP per QALY gained.

## Discussion

Our study is the largest prospective study on PADs including people living with mental illness who were compulsorily admitted during the past year. It is also the first to evaluate the costs and benefits of peer worker–facilitated PADs for the management of a patient’s mental disorder. In our study, for the overall population, PW-PAD was strictly dominant. The intervention was associated with a higher number of QALYs at a lower cost compared to usual care.

PW-PAD was associated with a significant decrease in total psychiatric inpatient days/nights and with a significant improvement in compulsory admissions, with no significant reduction in the rate of overall psychiatric admissions (Tinland *et al*., 2022). Our hypothesis is that, rather than preventing psychiatric hospital admissions, PADs may reduce compulsory admissions by making participants more willing to consider voluntary admission when a crisis occurs. The economic analysis goes further and shows, based on a detailed measure of health service utilization and cost analysis, that the use of PW-PAD reduces the overall length of stay and costs compared to usual care. Our findings are consistent with nonrandomized studies showing improvement in length of stay and costs associated with a shift from coercive measures (Dimitri *et al*., 2018, Kallert *et al*., 2008; McLaughlin *et al*., 2016, Salias and Fenton, 2000).

Few multicentre and randomized controlled studies have evaluated the effectiveness of advance directives in psychiatry, none incorporating facilitation by peer workers (Henderson *et al*., 2004; Molyneaux *et al*., 2019; Papageorgiou *et al*., 2002; Ruchlewska *et al*., 2014; Swanson *et al*., 2006; Thornicroft *et al*., 2013). Of these, only two had assessed their economic outcomes (Barrett *et al*., 2013; Flood *et al*., 2006). Flood *et al*. could not find any differences in admissions or total costs per participant over a 15-month follow-up. Similarly, Barrett *et al*. could not find any differences in compulsory admissions or total societal costs per participant over an 18-month follow-up. Neither of these studies found significant intervention effects on inpatient stays (i.e., number of nights). Our results show that the French participants spent considerable amount of time in hospital, regardless of the group, compared with those from UK studies with the same inclusion criteria. As an example, over 1 year, participants in both French study groups spent, on average, 2.5 times more days in hospital than participants in Barret’s UK-based study. The initial lengthy hospitalization duration, combined with its sharp fall over 1 year, explains the majority of our observed cost savings. This is consistent with the conclusion of Burns’ meta-analysis, which showed that interventions that reduce hospital admissions (in his paper. it is case management) are more effective when participants are high consumers of hospital care at baseline (Burns *et al*., 2007). Differences between the interventions could also explain these results: PW-PAD differ from Flood and Barret’s intervention in that they are PADs, and peer workers facilitate them. On the one hand, PADs are unique tools to promote self-determination (Elbogen *et al*., 2007), and part of the results could be due to this law-oriented form; on the other hand, peer workers’ involvement in healthcare has been shown to be associated with improvements in quality of life, self-efficacy, hope and empowerment (e.g., Fuhr *et al*., 2014; Lloyd-Evans *et al*., 2014; Mahlke *et al*., 2017; Vayshenker *et al*., 2016), which is consistent with our results. Unfortunately, our study does not allow us to identify the relative role of each ingredient in achieving good clinical or economic outcomes. PW-PAD is a complex intervention where the meeting with a peer worker and crisis reflection likely interact with each other.

Over the follow-up period, participants in the PW-PAD group gained more health utilities than their counterparts in the control group, with similar EQ5D-3L scores at baseline and 6 months. This finding could suggest a learning period with PW-PAD, with the effects in terms of quality of life appearing only after the first 6 months following the initiation of the advance directives, suggesting that this could be maintained in the long term. Further investigations are needed to understand the factors that influence the early benefits of the intervention and to help inform decision-making.

Even when the PW-PAD intervention was considered to cost double, the intervention still remained dominant compared to usual care. In fact, regardless of the variations considered in cost parameters, the PW-PAD group remained dominant, presenting a 100% chance of being cost-effective at small WTP thresholds for mental health programmes.

Such results are important because they inform decision makers and, perhaps more critically, they contribute to international guidelines on the economic efficiency of programmes in mental health. Indeed, cost analyses offer a perspective for policy changes. The PW-PAD is an intervention, which can be implemented in a fairly straightforward manner and can be very quickly cost-effective for the healthcare system.

### Strengths and limitations

The strengths of our study lie in our design and methodology. First, this research was deployed through seven psychiatric facilities, each with several services. The wide range of practices across these services reflects the diversity of practice in France. Between and within countries and regions, large variations have been found between services in rates of inpatient admissions and compulsion (Gandré *et al*., 2018b, 2018a; Hofstad *et al*., 2021; Weich *et al*., 2017), and these variations are not yet fully understood by the scientific literature (Rugkåsa, 2017). The diversity of participating centres, associated with broad inclusion criteria and limited exclusion criteria, enhances our confidence in the generalizability of our results. Second, we captured a measure of benefit expressed in terms of participant’s quality of life rather than simply a measure of resource use. Our approach is in accordance with international guidelines and avoids the double counting of resource use in both the denominator and numerator of the ICER (Neumann *et al*., 2016). Third, resource use was based on the data collected from hospital-based databases that captured the entire hospital pathway associated with the management of mental illness.

This trial had several limitations. First among them is the short follow-up period. Given the short-term nature of PAD fulfilment, the uncertainty surrounding long-term use and long-term consequences on health utility of PADs and the limited impact of PADs on survival, the 12-month randomized controlled trial timeline was deemed appropriate to capture most of the relevant costs and benefits associated with the intervention. Most of the incremental cost of PW-PAD is spent in the first 3 months, so this intervention might become even more cost-effective after 1 year if participants receiving the intervention continue to improve more than those receiving usual care without additional costs.

Second, we assumed the same costs for compulsory and standard hospitalizations. We may have underestimated the costs in the control group, although this reinforces the conservative costing approach. The accounting costs provided by French health agencies for hospitalization are not differentiated between compulsory or freely admitted patients, whereas compulsory hospitalization appears more expensive, especially at the beginning, often requiring more staff and special facilities such as isolation rooms (Flood *et al*., 2008; LeBel and Goldstein 2005). With 32% more compulsory admissions in the control group, total costs would be even higher if the associated containment and seclusion costs had been valued. At the same time, we did not account for the impact of discharge to the community on people other than the study patient (e.g., family or partners providing care to the patient) or measure costs of pharmaceuticals, the latter being assumed to be higher at entry in compulsory admission (Brown *et al*., 2010).

Finally, discontinuation rates were around one-third in both groups. Unfortunately, the COVID-19 pandemic generated a crisis that suddenly widened the gap in access to healthcare, especially for vulnerable populations. Our attrition rates were mainly due to lost to follow-up in periods of lockdown.

## Conclusions

Among people living with mental illness, the elaboration of PADs with the support of a peer worker was associated with a significant improvement in health utility in parallel to cost-savings over a 12-month period. These findings support a national-scale promulgation of this type of intervention and an implementation study of these tools to measure the level of adoption in common practice and to identify barriers and facilitators.

## Data Availability

Not all the data are freely accessible because no informed consent was given by the participants for open data sharing, but we can provide the data used in this study to researchers who want to use them, following approval by the ethics committee of the Aix Marseille University.

## Supplementary material

For supplementary material accompanying this paper visit http://doi.org/10.1017/S2045796023000197. click here to view supplementary material
