# Supplementary material for: Psychiatric advance directives facilitated by peer workers among people with mental illness: economic evaluation of a randomized controlled trial (DAiP study)
Source: Epidemiol Psychiatr Sci. 2023 Apr 25;32:e27. doi: 10.1017/S2045796023000197 (PMC10130836; doi:10.1017/S2045796023000197)
Supplement: Supplementary file 1 [file S2045796023000197sup001.docx]

**APPENDIX**

**Title** : Psychiatric advance directives facilitated by peer workers among people with mental illness: economic evaluation of a randomized controlled trial (DAiP study)

**Pages**: 11

**Tables**: 3

Table S1: Unit costs considered in the DAiP trial

Table S2: Total hospital admissions and length of stay in both compulsory and voluntary settings

Table S3: Results on complete data

**Figures: 3**

Figure S1: Outcomes regarding psychiatric advance directives (PADs) for all participants (N=394)

Figure S2: CONSORT Flow Diagram in the DAIP trial

Figure S3: Cost-effectiveness acceptability curve (CEAC) indicating the probability of cost-effectiveness for different thresholds of willingness-for-pay (€) per unit of effect (QALY) gained

**PW-PAD – English version**

**Table S1: Unit costs considered in the DAiP trial**

| **Category** | **Type** | **Unit costs (in Euros)** | **Sources** |
| --- | --- | --- | --- |
| ***Inpatient costs*** |  |  |  |
| Hospitalizations - psychiatric hospital (public) | Day care | 231.67/day | ATIH – Scan santé  https://www.scansante.fr 2019 |
|  | Partial time | 229.00/day |  |
|  | Complete | 369.68/day |  |
|  | Consultation | 104.03 |  |
| Hospitalizations – medical (public) | Complete | 497.28/day | ATIH – Scan santé  https://www.scansante.fr 2019 |
|  | Consultation | 92.76 |  |
| Supplemental fees | Intensive care unit | 1 888.81 | ATIH – Scan santé  https://www.scansante.fr 2019 |
|  | Palliative care | 1 117.94 |  |
|  | Surveillance | 862.13 |  |
| Emergency Department visit | General medicine | 161.50 | Rapport Cours des Comptes 2019 |
|  | Psychiatry | 185.84 |  |
| Transport | Emergency ambulance | 933.80 | Rapport Sécurité Sociale 2017 |
|  |  |  |  |
| ***Ambulatory costs*** |  |  |  |
| Consultations | General practitioner | 25.00/visit | NGAP - www.ameli.fr |
|  | Psychiatrist | 46.70/visit | NGAP - www.ameli.fr |
|  | Other specialist | 25.00/visit | NGAP - www.ameli.fr |
|  |  |  |  |
| ***Intervention costs*** |  |  |  |
| Training | 2 half-day sessions per week for 6 weeks | 1215.00/pw |  |
| PW’s salary | Monthly amount (including charges) | 3 738.00/pw | Peer worker (pw) interwiew |
| Transport indemnity | Monthly amount | 75.00/pw |  |
|  |  |  |  |

PW: peer-worker. ATIH: *Agence technique de l'information sur l'hospitalisation* (Agency for Information on Hospital Care - AIHC). NGAP: *Nomenclature générale des actes professionnels* (Relative value scale of medical procedures)

**Table S2: Total psychiatric hospital admissions and length of stay in both compulsory and voluntary settings**

|  | **PW-PAD group** | **Control group** |  |
| --- | --- | --- | --- |
| **Psychiatric hospital resource use** | **N (%) or**  **mean (sd)** | **N (%) or**  **mean (sd)** | **P-value** |
| No. (%) of patients with at least one psychiatric admission | 70 (35.70) | 79 (39.90) | 0.392 |
| No. (%) of patients with at least one compulsory admission | 53 (27.00) | 79 (39.90) | 0.007 |
| Mean (sd) admissions per patient | 1.07 (2.04) | 1.44 (1.97) | 0.070 |
| Mean (sd) days at hospital | 45.4 (89) | 57.1 (88) | 0.026 |
| Mean (sd) days in compulsory admission | 20.4 (62) | 28.0 (72) | 0.002 |
| Mean (sd) days in voluntary admission | 25.0 (66) | 29.1 (62) | 0.147 |

PW-PAD: peer-worker facilitated psychiatric advance directive; sd: standard deviation.

**Table S3: Mean and incremental costs and QALYs for patients receiving PW-PAD versus usual care based on complete data**

|  | **PW-PAD group** | **Control group** |  |  |
| --- | --- | --- | --- | --- |
|  | **Mean (95%CI)** | **Mean (95%CI)** | **Mean change from baseline (95%CI)** | **P Value** |
| **Utilities** | 0.808 (0.778 – 0.839) | 0.763 (0.733 – 0.793) | 0.045 (0.002 – 0.088) | 0.039 |
|  | **Mean (95%CI)** | **Mean (95%CI)** | **Difference in mean (95%CI)** | **P Value** |
| **Costs** | 20 467 (16 307 – 24 626) | 25 346 (20 427 – 30 265) | -4 879 (-11 321 – 1 562) | 0.138 |

PW-PAD: peer-worker facilitated psychiatric advance directive; QALY: quality-adjusted life year; CI indicates confidence interval.

**Figure S1. Outcomes regarding psychiatric advance directives (PADs) for all participants (N=394)**

**
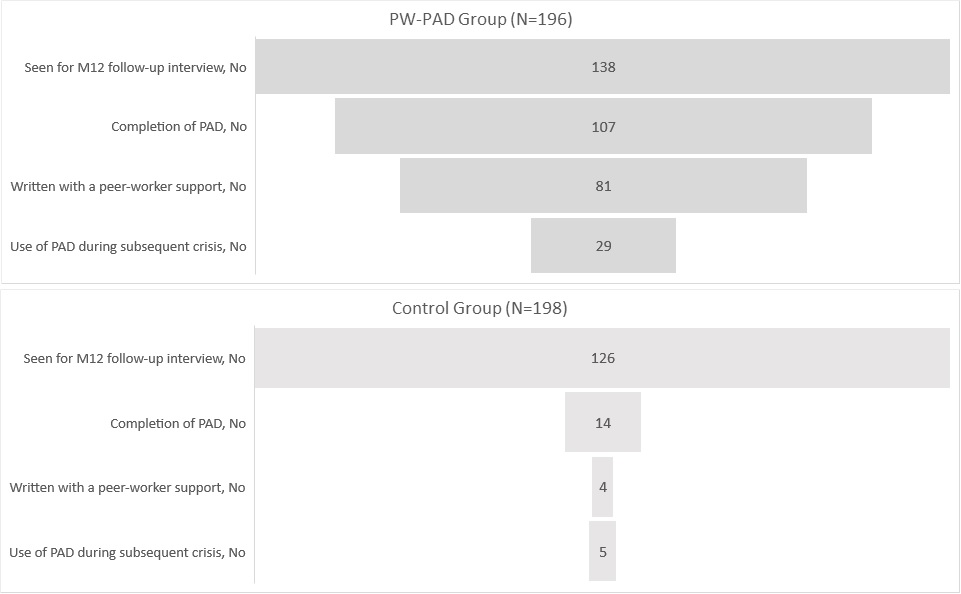
**

**Legend Figure S1:**

PAD: psychiatric advance directive; PW-PAD: peer-worker facilitated psychiatric advance directive.

**Figure S2: CONSORT Flow Diagram for the DAIP trial**

**473 Assessed for eligibility**

**from January 2019 to June 2020**

1. **Excluded by Research assistants**

48 Declined to participate (not interested, worried that drafting PADs may lead to a crisis)

14 Had no contact after reflection

10 Did not meet inclusion criteria

**401 Randomized individuals**

**200 Intervention group**

**201 Control group**

**Allocation**

1. **Excluded by Data board**

- 1 with Eligibility criteria not verified
- 2 withdrawals

1. **Excluded by Data board**

- 1 with Eligibility criteria not verified
- 3 withdrawals

**198 Control group**

**196 Intervention group**

**Follow-up**

**at M6**

198 patients with Primary outcome data

127 completed the interview

71 discontinued

- 1 Patient who died
- 69 Patients did not attend six-month follow-up
- 1 Withdrawal

196 patients with Primary outcome data

118 completed the interview

78 discontinued

- 0 Patient who died
- 78 Patients did not attend six-month follow-up
- 0 withdrawal

**Follow-up**

**at M12**

198 patients with Primary outcome data

139 completed the interview

60 discontinued

- 1 Patient who died
- 56 Patients did not attend 12-month follow-up
- 2 Withdrawals

196 patients with Primary outcome data

127 completed the interview

69 discontinued

- 0 Patient who died
- 68 Patients did not attend 12-month follow-up
- 1 withdrawal

198 analysed

in intention-to-treat analysis

196 analysed

in intention-to-treat analysis

**Intention-to-treat Analysis**

**Figure S3: Cost-effectiveness acceptability curve (CEAC) indicating the probability of cost-effectiveness for different thresholds of willingness-for-pay (€) per unit of effect (QALY) gained**


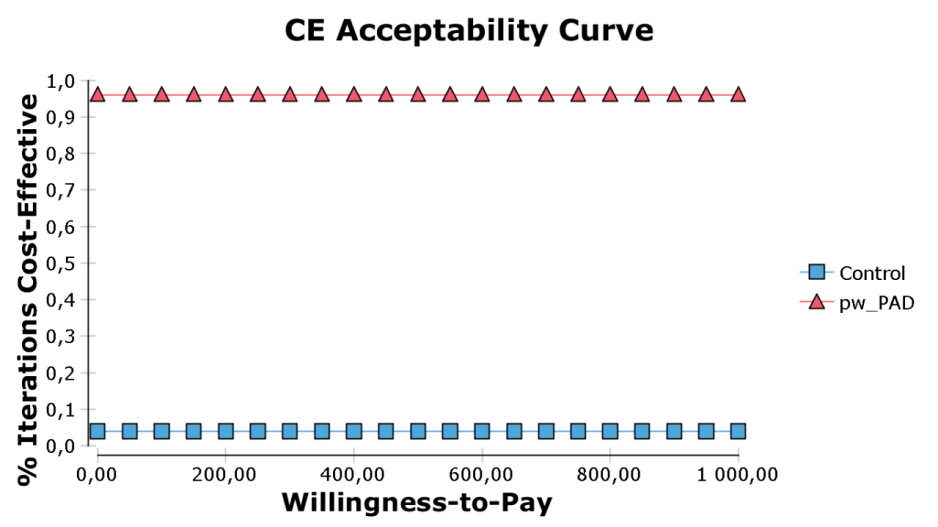


**Legend Figure S3:**

PW-PAD: peer-worker facilitated psychiatric advance directive; QALY: quality-adjusted life year; CE: Cost-effectiveness; CEAC: Cost-effectiveness acceptability curve.

The cost-effectiveness acceptability curve provides visual results of the probabilistic sensitivity analysis. CEAC presents the relative cost-effectiveness as a function of the ICER threshold (WTP). For each WTP value, the graph uses net benefits to determine the percentage of simulation iterations that favor each strategy. The percentages will increase for more effective strategies as the WTP increases.

## PW-PAD form – version in English

## Psychiatric Advance directives

*Person concerned and health care agent*

I, undersigned, ………………………………………………………………………………………….............……., born on….…/………/………. in ……………………………………......., designates as health care agent in accordance with the article L. 1111-6 of the Public Health Code:

Contact Type of relationship Function

| 1. M.-Mrs. |  |  |  |
| --- | --- | --- | --- |
| 1. M.-Mrs. |  |  |  |
| 1. Others |  |  |  |

To ensure the carrying out the of my following Psychiatric Advance Directives, with my consent at the time of writing.

It is understood that the Psychiatric Advance Directives are not binding on relatives and caregivers.

Done in …………… the ……/……./……./………, alone or with ……………………………………………

Name(s) : Signature(s) :

| - When I am competent to decide: | - When I am not competent to decide: |
| --- | --- |

*Warning signs*

1 – 3 –

2 – 4 –

New signs (« update ») : _________________________________________________________________________________________________________________________________________________________________________________________________________________________________

*What help me in case of crisis :*

1 – 3 –

2 – 4 –

*What doesn’t help me in case of crisis :*

1 – 3 –

2 – 4 –

*Signals of end of crisis, when it gets better:*

1 – 3 –

2 – 4 –

*In case of danger (auto or hetero agressivity)*

To do : Not to do :

- -

- -

- -

| Medication | Dosage | Effect | Note |
| --- | --- | --- | --- |
|  |  |  |  |
|  |  |  |  |
|  |  |  |  |
|  |  |  |  |

**I want to take a medication:**  YES □ NO □

*Medications that help me, that can be used*

*–* Article 1111.4 of the Public Health Code about the choice of treatment

| Medication | Dosage | Effect | Note |
| --- | --- | --- | --- |
|  |  |  |  |
|  |  |  |  |
|  |  |  |  |
|  |  |  |  |

*Medications that do not help me, that should not be used*

**I want to be accompanied to a care setting:** YES □ NO □

*Care settings I want to use (hospitals, services, teams)*

- Article 1110.8 of the Public Health Code on the choice of practitioner and health facility

- -

- -

- -

Remarks : …………………………………………………………………………………………………………………………………………………………………………………………………………………………………………………………………………………………………………………………………………………………………………………

*Care settings I don’t want to use :*

- -

- -

- -

Remarks: …………………………………………………………………………………………………………………………………………………………………………………………………………………………………………………………………………………………………………………………………………………………………………………………

*Health care professionals*

- I want to be supported by : - I don’t want to be supported by :

* *

* *

* *

*Other personal remarks, useful tips, notes…*

…………………………………………………………………………………………………………………………………………………………………………………………………………………………………………………………………………………………………………………………………………………………………………………………………………………………………………………………………………………………………………………………………………………………………………………………………………………………………………………………………………………………………………………………………………………………………………………………………………………………………………………………………………………………………………………………………………………………………………………………………………………………………………………………………………………………………………………………………………………………………………………………………………………………………………………………………………………………………………………………………………………………………………………………………………………………………………………………………………………………………………………………………………………………………………………………………………………………………………………………………………………………………………………………………………………………………………………………………………………………………………………………………………………………………………………………………………………………………………………………………………………………………………………………………………………………………………………………………………………………………………………………………………………………………………………………………………………………………………………………………………………………………………………………………………………………………………………………………………………………………………………………………………………………………………………………………………………………………………………………………………………………………………………………………………………………………………………………………………………………………………………………………………………………………………………………………………………………………………………………………………………………………………………………………………………………………………………………………………
